# Impact of High Glucose and Proteasome Inhibitor MG132 on Histone H2A and H2B Ubiquitination in Rat Glomerular Mesangial Cells

**DOI:** 10.1155/2013/589474

**Published:** 2013-04-30

**Authors:** Chenlin Gao, Guo Chen, Li Liu, Xia Li, Jianhua He, Lan Jiang, Jianhua Zhu, Yong Xu

**Affiliations:** Department of Endocrinology, Affiliated Hospital of Luzhou Medical College, Luzhou 646000, China

## Abstract

*Background*. Hyperglycemia plays a pivotal role in the development of diabetic nephropathy (DN) and may be related to epigenetic metabolic memory. One of the most crucial epigenetic mechanisms is histone modification, which is associated with the expression of a fibrosis factor in vascular injury. *Aim* .In this study, we investigated the ubiquitination of histones H2A and H2B to explore the epigenetic mechanisms of DN. *Materials and Methods*. The GMCs were cultured as follows: normal group, high glucose group, mannitol group, and intervention group. After 12 hr, 24 hr, and 48 hr, histones ubiquitination, transforming growth factor-*β* (TGF-*β*), and fibronectin (FN) were measured using WB, RT-PCR, and IF. *Result*. High glucose can induce the upregulation of FN. H2A ubiquitination in GMCs increased in high glucose group (*P* < 0.01), whereas it decreased significantly in intervention group (*P* < 0.05). In contrast, H2B ubiquitination decreased with an increasing concentration of glucose, but it was recovered in the intervention group (*P* < 0.05). Expression of TGF-*β* changed in response to abnormal histone ubiquitination. *Conclusions. *The high glucose may induce H2A ubiquitination and reduce H2B ubiquitination in GMCs. The changes of histone ubiquitination may be due in part to DN by activating TGF-*β* signaling pathway.

## 1. Introduction

Diabetic nephropathy (DN) is one of the most devastating microvascular complications of diabetes, which remains the most common cause for end stage renal disease (ESRD). The prevalence of diabetes and the patients suffering from diabetic microvascular complications is increasing worldwide [1]. Nearly one-third of patients with diabetes develop nephropathy, and early diagnosis is critical in preventing long-term kidney loss [2]. However, the mechanisms that cause DN have not been completely clarified, and the treatment options are limited.

Hyperglycemia plays a pivotal role in activating various inflammatory pathways in the development and progression of DN. It induces the fibrotic factor transforming growth factor-*β* (TGF-*β*) and fibronectin (FN), the renin-angiotensin-aldosterone system (RAAS), and advanced glycation end products both directly and via gene transcription, which leads to thickening of the glomerular and tubular basement membranes, progressive accumulation of extracellular matrix (ECM) proteins, interstitial fibrosis, and glomerulosclerosis [3–6]. FN is one of the main ingredients of ECM and an important symbol of cell injury. The upgrade expression of FN will eventually lead to the development of diabetic nephropathy. Clinical trials have reported that strict glycemic control reduces the progression of diabetic complications over time. Diabetic complications, including chronic kidney disorders such as DN, have been shown to persist and progress even after glucose control has been achieved, possibly as a result of prior episodes of hyperglycemia that are considered the epigenetic metabolic memory [7]. Preliminary work using endothelial cells has shown that transient episodes of hyperglycemia can induce changes in gene expression that are dependent on the modification of histone tails (i.e., methylation) [8]. The persistence of such modifications has not been fully explained. Additional studies regarding the epigenetic mechanisms involved are necessary to provide valuable new insights into the pathology of DN. 

Posttranslational modifications of the aminoterminal tails of nucleosomal histones, including acetylation, methylation, ubiquitination, phosphorylation, and sumoylation, play key roles in modulating the chromatin structure and gene transcription that have been implicated in regulating the metabolism of diabetic complications [9]. The modification of histones by ubiquitination is a prominent epigenetic mark that may influence changes in gene expression and involves a variety of chromatin-based events, such as gene silencing and repair of DNA damage [10]. The majority of histone ubiquitination occurs on chromatin by the addition of a single ubiquitin molecule via isopeptide linkage to a specific lysine residue on the C-terminal tail of histones H2A and H2B. To a lesser extent, histones H1, H3, and H4 can be ubiquitylated in vivo, and ubiquitination of different histones has distinct functions [11]. However, the effects of histone ubiquitination on DN are unclear.

Recent research has indicated that histone modification is directly or indirectly related to diabetic attacks [12–15]. Histone acetylation can activate the TGF-*β* signaling pathway, which plays an important role in DN renal fibrosis. Similarly, DN is associated with increased renal H3K9 and H3K23 acetylation, H3K4 dimethylation, and H3 phosphorylation at serine 10, which enhances chromatin unfolding and gene expression [16, 17]. To date, it is unknown whether histone ubiquitination is involved in interstitial fibrosis and glomerulosclerosis in DN or whether the effects of hyperglycemia on such epigenetic events can be mediated through TGF-*β* signaling pathways. MG132, a proteasome inhibitor, is suggested to attenuate hypertension-induced cardiac remodeling and dysfunction by downregulating the levels of TGF-*β*_1_ [18]. Whether ubiquitin proteasome inhibitors can inhibit renal fibrosis which was followed by activation of the TGF-*β* signaling pathway in diabetic nephropathy remain unclear. So, additional research to develop new treatments for DN is necessary. In this study, we evaluated the influence of high glucose on the induction of histone H2A ubiquitination, reduced histone H2B ubiquitination in GCMs, and changes in the expression of TGF-*β* followed by abnormal histone ubiquitination. MG132, which acts as a ubiquitin proteasome inhibitor, may prevent the alterations in H2A and H2B ubiquitination induced by high glucose.

## 2. Materials and Methods

### 2.1. Cell Culture

Cell culture media and fetal bovine serum were purchased from Hyclone (USA). Rat glomerular mesangial cells (HBZY-1) were purchased from the Preservation Center at Wuhan University and maintained in low glucose DMEM with 10% fetal bovine serum at 37°C and 5% CO_2_. GMCs were used between the 2nd and 5th passages for all experiments and randomly divided into the following six groups:

- the normal control group (NC group), with medium containing 5.6 mmol/L glucose,
- the 10 mmol/L glucose group (HG1 group), with medium containing 10 mmol/L glucose,
- the 20 mmol/L glucose group (HG2 group), with medium containing 20 mmol/L glucose,
- the 30 mmol/L glucose group (HG3 group), with medium containing 30 mmol/L glucose,
- the osmotic pressure group as a control (OP group), with medium containing 5.6 mmol/L glucose + 22.4 mmol/L mannitol,
- the MG132 intervention group (MI group), with medium containing 30 mmol/L glucose + 1 *μ*mol/L MG132. MG132 was added to the culture medium to block the ubiquitination of histone.

Each group had been cultured for 12 hr, 24 hr, and 48 hr.

### 2.2. Protein Extraction and Western Blot

Total proteins were isolated from GMCs using a total protein extraction kit (Kaiji, Shanghai, China). The protein concentration was determined using BCA analysis (Beyotime, Shanghai, China). Western blotting was performed as previously described [19]. Immunoblot analysis was performed using anti-ubiquityl-histone H2A (mouse, 1 : 1000; Millipore, USA), anti-ubiquityl-histone H2B (rabbit, 1 : 1000; CST, USA), and anti-GAPDH and actin (rabbit, 1 : 1000; Beyotime, Shanghai, China). Horseradish peroxidase-conjugated secondary antibodies (anti-rabbit and anti-mouse) were obtained from the Beyotime Institute of Biotechnology, Shanghai, China. Proteins were detected using the enhanced chemiluminescence system and ECL Hyperfilm (Millipore, USA).

### 2.3. RNA Extraction and Semiquantitative PCR

Total RNA was extracted from GMCs using an RNA extraction kit (Tiangen Biotech, Beijing, China). A total of 500 ng RNA was reverse transcribed at a final volume of 20 *μ*L using a Takara RNA PCR kit (Baoshengwu, Dalian, China). Four microliters of cDNA were amplified in a gradient thermal cycler (Eppendorf, Germany) using the PCR Master Mix (Baoshengwu, Dalian, China). The results were determined using a UV transilluminator and normalized to glyceraldehyde 3-phosphate dehydrogenase (GAPDH) gene expression. The primer sequences were as follows: histone H2A ubiquitination (uH2A), forward, 5′-GCA CCC TGA CCT TGC CTA T -3′, reverse, 5′-CCT TCC CAG ACT CCA CCA T-3′, histone H2B ubiquitination (uH2B), forward, 5′-CGC CTG GCT CAT TAC AAC-3′, reverse, 5′-CTT GGT TTC CGA CA-3′, transforming growth factor-*β* (TGF-*β*), forward, 5′-ATG GTG GAC CGC AAC AAC-3′, reverse, 5′-GAG CAC TGA AGC GAA AGC-3′, FN, forward, 5′- TGC CGA ATG TAG ATG AGGA -3′, reverse, 5′-AAA TGA CCA CTG CCA AAGC -3′, and GAPDH, forward, 5′-CCT CAA GAT TGT CAG CAA T -3′, reverse, 5′-CCA TCC ACA GTC TTC TGA GT-3′.

### 2.4. Immunofluorescence

Cells were grown on coverslips in 6-well plates. After overnight adherence, the cells were treated with media containing high glucose, mannitol, and the MI media for 24 hr. The cells were fixed in 4% paraformaldehyde (Pierce) and permeabilized in 0.2% Tween 20 (Sigma) for 10 min after being washed briefly with PBS. The cells were blocked with 5% goat serum for 1 hr at room temperature and incubated overnight with primary antibodies followed by washes with PBS. The cells were incubated for 40 min with the appropriate secondary antibody conjugated to the FITC fluorescent dye. DAPI (4′,6′-diamino-2-phenylindole) was used to stain the nucleus in the cells. The coverslips were washed and mounted onto slides using fluorescent mounting medium (Beyotime, Shanghai, China). The control cells were incubated without a primary antibody. Images were taken with a DMIRE_2_ laser scanning confocal microscope (Leica, Germany). 

### 2.5. Statistics

All experimental data were obtained from three independent experiments performed in triplicate. Data were expressed as the mean ± SD ($\bar{x}\;\pm \;s$), which were analyzed using SPSS 11.5 statistical software. Statistical differences were calculated using one-way analysis of variance (one-way ANOVA) to compare more than two groups, followed by the LSD test for multiple comparisons. *P* < 0.05 was defined as statistically significant.

## 3. Results

**Figure 1:**
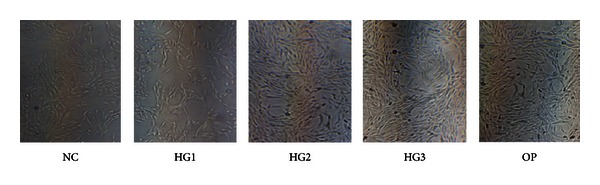
Cell picture in each group of GMCs by inverted phase contrast microscope (×100). No significant changes in cell morphology.

**Figure 2:**
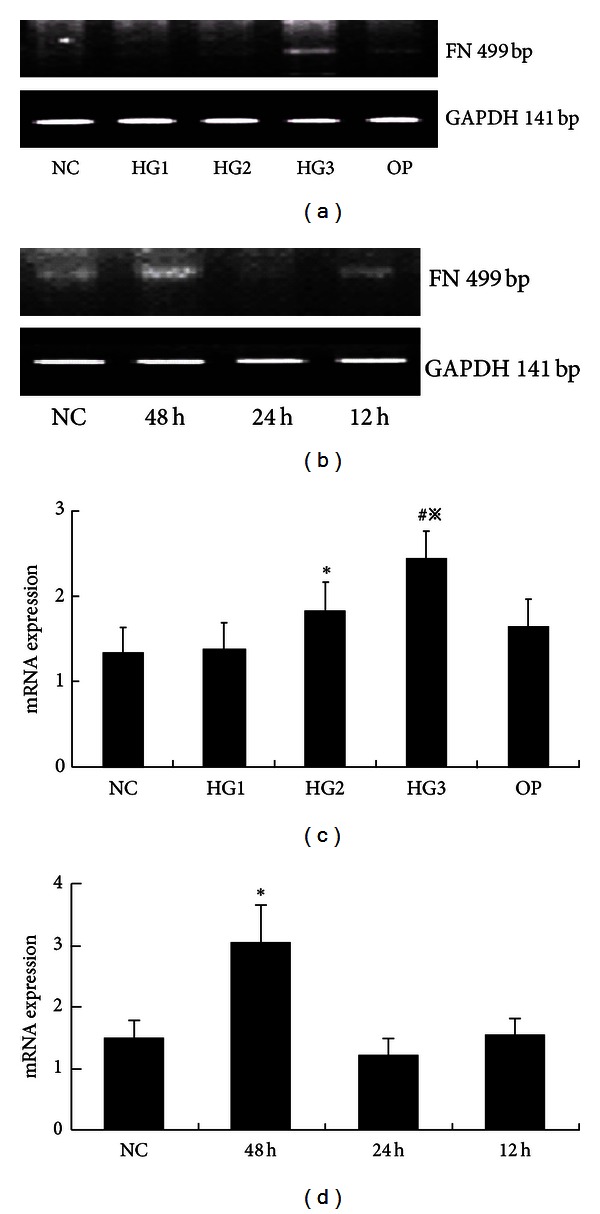
The mRNA levels of FN in each group of GMCs. (a) RT-PCR strip chart for different concentrations of glucose. FN mRNA increased in the high glucose group, especially in HG3 group. (c) The corresponding relative gray value statistics graph of the mRNA level. *P < 0.05 versus NC group, ^#^ *P* < 0.01 versus NC group, and ^*※*^ *P* < 0.05 versus HG2 group. (b) RT-PCR strip chart for different times. The expression of FN mRNA increased over time. (d) The corresponding relative gray value statistics graph of the mRNA level. *P < 0.01 versus NC group.

The cellular morphology in different glucose culture medium is not significantly changing (Figure 1), but the expression of FN increased in the high glucose group over time, especially in HG3 group for 48 hr (Figure 2).

**Figure 3:**
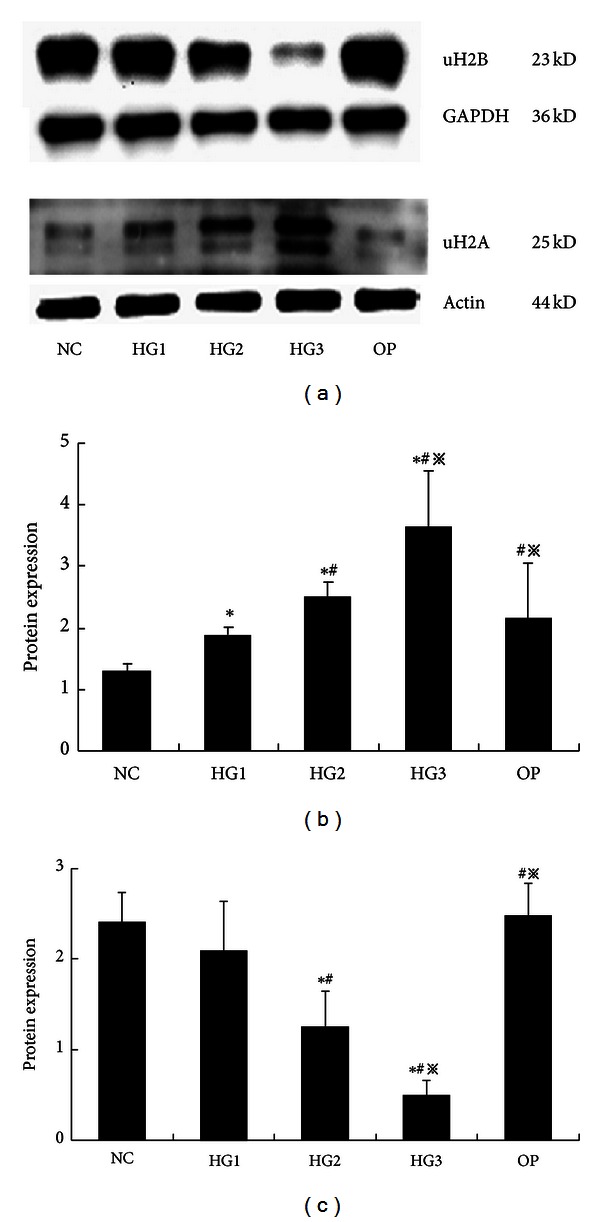
The expression of histone H2A and H2B ubiquitination in each group of GMCs by Western blot (48 hr). (a) uH2A and uH2B proteins at different glucose concentrations and high osmotic pressure at 48 hr: uH2A increased in the high glucose group and uH2B decreased as the glucose concentrations increased; they had the most significant changes in the HG3 group, but there were no apparent differences between the NC group and the OP group. (b) The gray graph shows the relative statistical values of uH2A for each group. The expression of the uH2A increased in the high glucose group, especially, in the HG3 group. *P < 0.01 versus NC group, ^#^ *P* < 0.03 versus HG1 group, and ^*※*^ *P* < 0.05 versus HG2 group. (c) The gray graph shows the relative statistical values of uH2B for each group. The expression of the uH2B proteins decreased by concentration dependency, obviously in HG3 group. *P < 0.01 versus NC group, ^#^ *P* < 0.03 versus HG1 group, and ^*※*^ *P* < 0.05 versus HG2 group.

After 48 hr in culture, Western blot analysis showed low H2A ubiquitin expression in the NC group. Expression was higher in the high glucose group compared to the NC group (*P* < 0.01) in a concentration dependent manner. The strongest expression was in the 30 mmol/L high glucose group (*P* < 0.01). In contrast, H2B ubiquitination expression was strong in the NC group. There were no significant differences in H2B ubiquitination expression in the 10 mmol/L high glucose group compared to the NC group (*P* = 0.327). Expression was lower in the 20 and 30 mmol/L high glucose groups compared to the NC group (*P* < 0.01) and was the weakest in the 30 mmol/L high glucose group. There were no differences between the OP and NC groups regarding the ubiquitination of H2A and H2B (*P* > 0.05) (Figure 3). 

**Figure 4:**
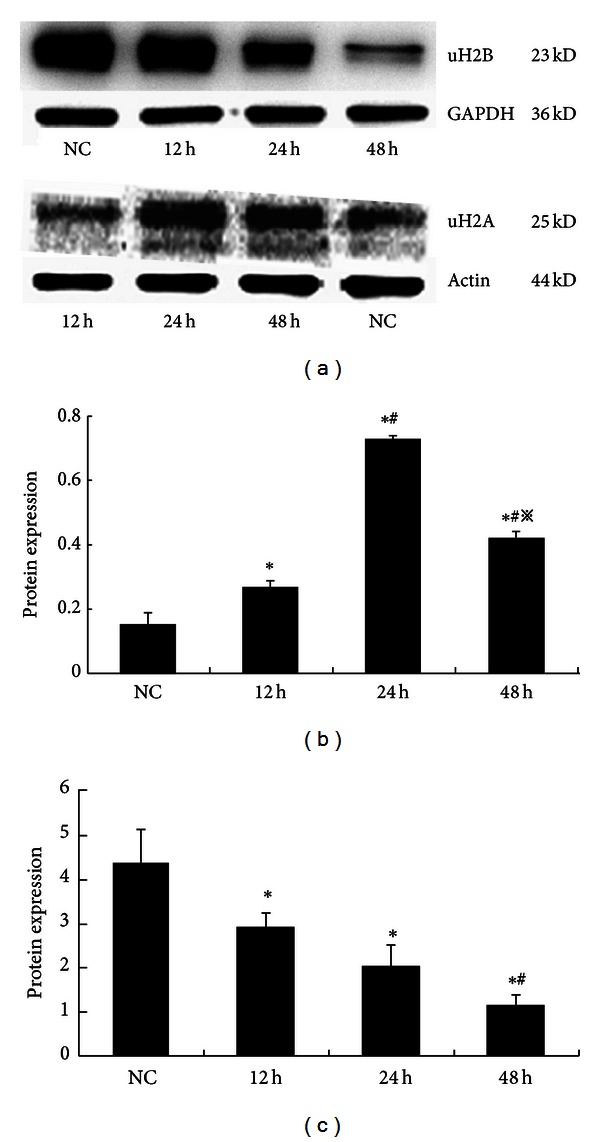
The expression of histone H2A and H2B ubiquitination in GMCs induced by 30 mmol/L glucose at various times determined using Western blot. (a) Compared to the NC group, uH2A expression increased after stimulation with 30 mmol/L glucose after 24 hr, but the uH2B expression decreased over time. (b) The gray graph shows the relative statistical values for uH2A protein expression at various time points for each group. The expression of uH2A was increased at 24 h sharp. *P < 0.01 versus NC group, ^#^ *P* < 0.05 versus 12 h, and ^*※*^ *P* < 0.05 versus 24 h. (c) The gray graph shows the relative statistical values for uH2B protein expression at various time points for each group. And the change of uH2B expression was a time dependent reduction. *P < 0.01 versus NC group, ^#^ *P* < 0.05 versus 12 h, and ^*※*^ *P* < 0.05 versus 24 h.

**Figure 5:**
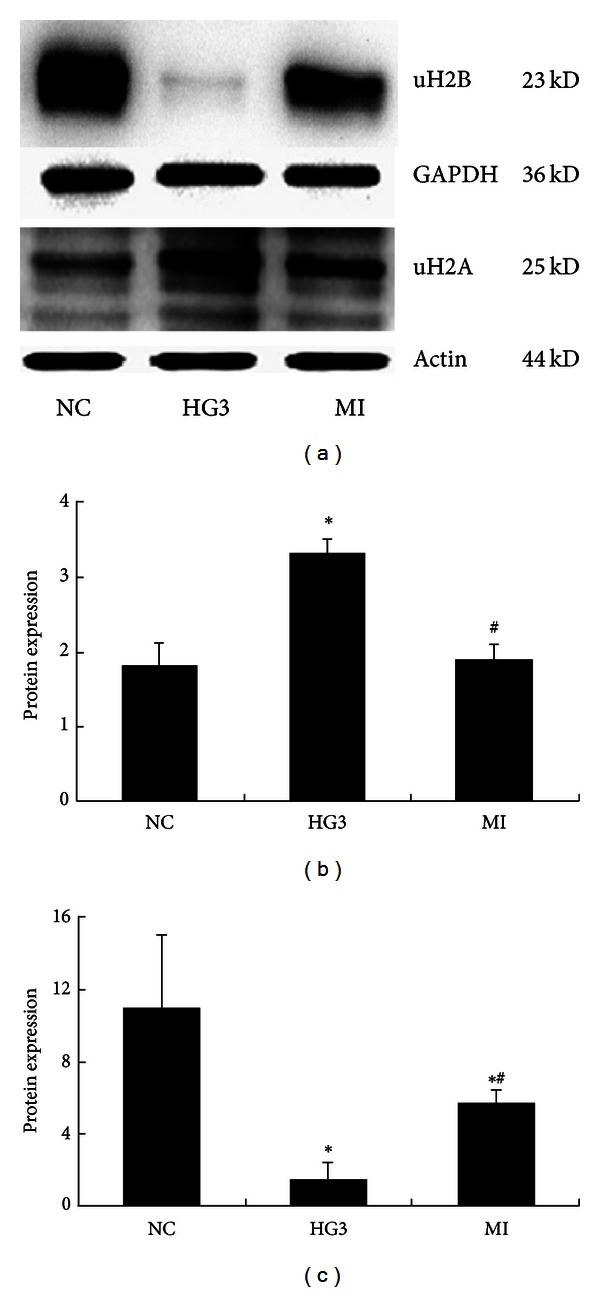
The expression of histone H2A and H2B ubiquitination after intervention with MG132 determined using Western blot (uH2A for 24 hr and uH2B for 48 hr). (a) Western blot strip chart. (b) The gray graph shows the relative statistical values of uH2A for each group. Compared with the NC group, the expression of the uH2A protein was significantly increased in the HG3 group. After MG132 intervention, the protein expression of uH2A decreased. *P < 0.01 versus NC group and ^#^ *P* < 0.05 versus 1 HG3 group. (c) The gray graph shows the relative statistical values of uH2B for each group. Compared with the NC group, the expression of the uH2B protein was significantly decreased in the HG3 group. And after MG132 intervention, uH2B relative to the normal group changed. *P < 0.01 versus NC group and ^#^ *P* < 0.05 versus 1 HG3 group.

**Figure 6:**
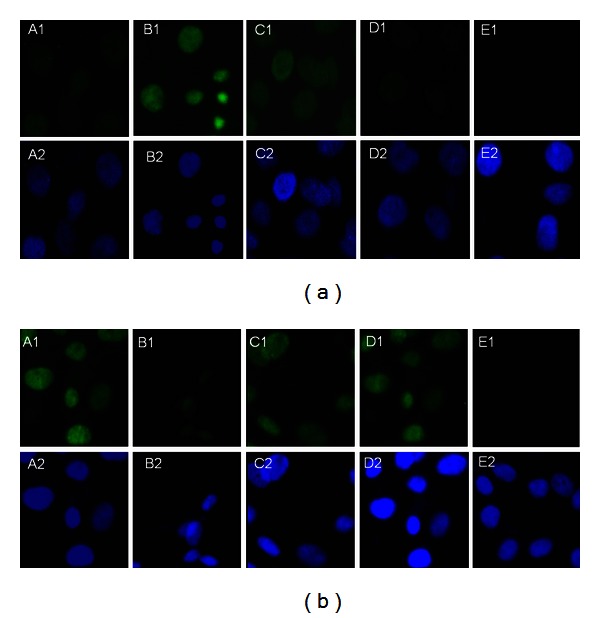
The expression of histone H2A and H2B ubiquitination in GMCs by immunofluorescent staining and laser scanning confocal microscopy applications. A1, NC group, B1, HG3 group, C1, MI group, D1, OP group, E1, negative control, and A2, B2, C2, D2, and E2 represent the corresponding groups of nuclear stains. (a) uH2A protein and (b) uH2B protein. uH2A and uH2B proteins were detected in the nucleus as green fluorescence overlapping with the blue fluorescence emitted by the nuclear stains DAPI. The uH2A protein was not detected in the NC group, but it was prominent in the HG3 group. The protein was strongly detected in the MG132 intervention group, which did not significantly change as a result of high osmotic pressure. The results for the uH2B protein were in contrast to the uH2A results.

The expression of the uH2A protein increased at various time intervals in the HG3 group, particularly after 24 hr in culture. The increased expression of uH2A at various time points was statistically significant (*P* < 0.01). Expression of the uH2B protein was significantly reduced in a time dependent manner (*P* < 0.01) (Figure 4). After MG132 intervention, the expression of uH2A was significantly decreased compared to expression in the 30 mmol/L glucose group (*P* < 0.05). In contrast, the expression of uH2B was recovered in the MI group compared to the HG3 group (*P* < 0.05) (Figure 5). The same results were detected using cell immunofluorescent staining and laser scanning confocal microscopy applications (Figure 6).

**Figure 7:**
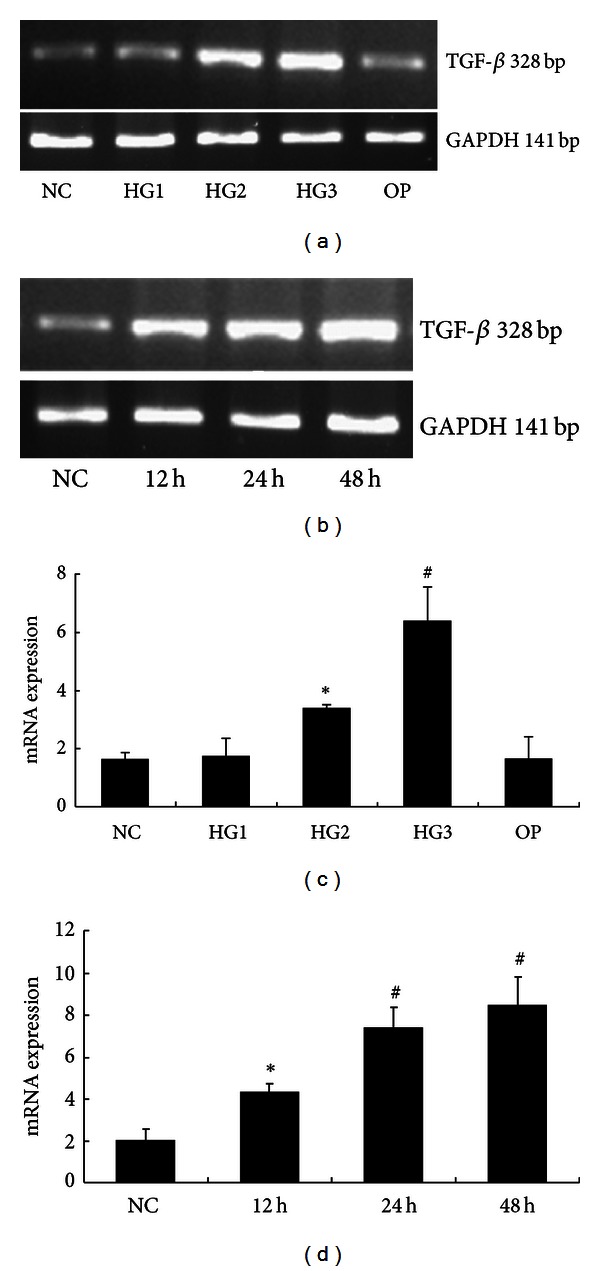
The mRNA levels of TGF-*β* in each group of GMCs. (a) RT-PCR strip chart for different concentrations of glucose. (c) The relative gray value statistics graph of the mRNA level for different concentrations of glucose. (b) and (d) The RT-PCR strip chart and the relative gray value statistics graph of the mRNA level for different times. *P < 0.05 versus NC group and ^#^ *P* < 0.01 versus NC group.

**Figure 8:**
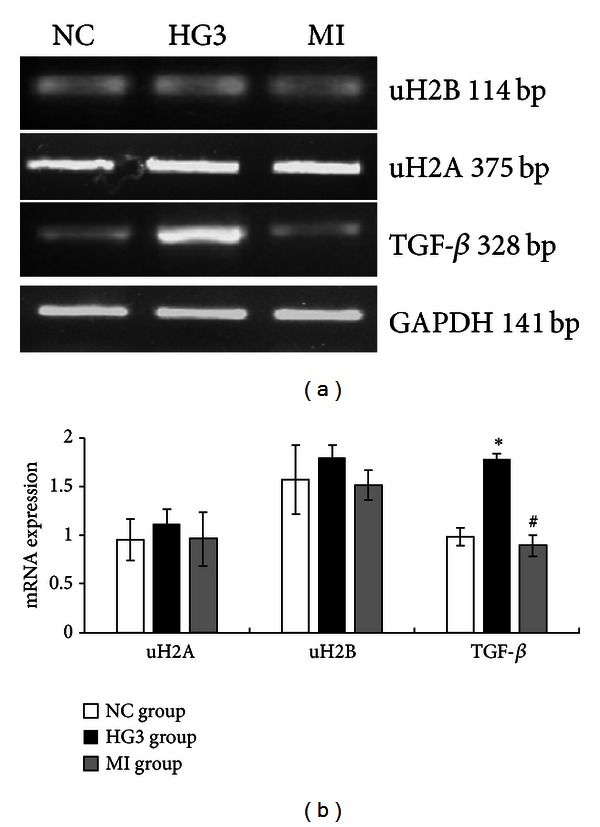
The mRNA levels of uH2A, uH2B, and TGF-*β* in GMCs induced by high glucose and inhibited by MG132. (a) RT-PCR strip chart. (b) The relative gray value statistics graph of the mRNA level. Compared with the NC group, the mRNA levels of uH2A and uH2B were not different between the groups. The expression of TGF-*β* mRNA was greatly increased in the HG3 group and decreased in the MI group compared to the HG3 group. *P < 0.01 versus NC group and ^#^ *P* < 0.01 versus 1 HG3 group.

The expression of TGF-*β* mRNA increased in the high glucose group compared to the NC group (*P* < 0.01) in a concentration and time dependent manner (Figure 7). And it decreased along with the normalization of uH2A and H2B protein expression (*P* < 0.05), although the mRNA levels of uH2A and H2B were not statistically different in each group (Figure 8). 

## 4. Discussion

Hyperglycemia plays an important role in the development and progression of DN, which can induce the expression of FN and cause cell injury, but the DNA sequence changes cannot solely explain the heritable patterns of gene expression. However, hyperglycemic memory may explain why intensive glucose control has failed to improve cardiovascular outcomes in patients with diabetes, although the molecular mechanisms of this phenomenon remain to be elucidated. The current study found histone modifications can change the state of evacuation and aggregation in chromatin by affecting compatibility between the histones and double-stranded DNA and influencing the affinity of transcription factors for structural gene promoters, which can regulate the expression of genes [20]. Recent studies have shown that diabetes-induced epigenetic changes can affect gene expression in vascular endothelial cells and vascular smooth muscle cells and long-lasting changes in epigenetic modifications at key inflammatory gene promoters following exposure to diabetic conditions [7, 21]. Histone acetylation attenuates epidermal growth factor signaling, which has a key role in the development of DN, and genome-wide studies have shown cell-type specific changes in histone methylation patterns under conditions of DN [22, 23]. And in diabetic retinopathy, histone acetylation was significantly increased in retinas from diabetic rats and contributed to the hyperglycemia-induced upregulation of proinflammatory proteins and thereby to the development of diabetic retinopathy [24]. In DN, histone acetylation, specific histone acetyl transferases, and histone deacetylases significantly enhanced TGF-*β*1-induced gene expression in rat mesangial cells and in glomeruli from diabetic mice and augmented glomerular dysfunction linked to diabetic nephropathy [25]. Histone methylation has also gained much attention as potential molecular mechanisms underlying metabolic memory and DN. The specific Set7 methyltransferase is the best characterised lysine enzyme, which showed high expression in DN. Furthermore, the contribution of Set7 to the aetiology of diabetic complications may extend to other transcriptional events through methylation of nonhistone substrates [26, 27]. However, little is known about histone ubiquitination in diabetic nephropathy. In this study, we found high glucose may cause cell damage, induce the ubiquitination of histone H2A, and reduce the ubiquitination of histone H2B in GMCs.

The results indicate that histone H2A and H2B ubiquitination may be involved in the development and progression of DN as an epigenetic mechanism. Although the mechanisms of action vary for different histones, ubiquitination of histone H2A K119 may induce DN and ubiquitination of histone H2B K120 has been shown to delay the onset of DN.

TGF-*β* has been implicated in various human disorders, including vascular and renal diseases, and is a primary fibrotic factor. Diabetic nephropathy (DN) is a chronic renal complication characterized by thickening of the glomerular and tubular basement membranes and progressive accumulation of extracellular matrix (ECM) proteins, such as type I and type IV collagens, fibronectin, and laminin in the tubular interstitium and mesangium. TGF-*β* increases ECM accumulation and plays a major role in the development of chronic renal diseases through the induction of a downstream effector, which is a connective tissue growth factor, and by decreasing matrix degradation through the inhibition of proteases or activation of protease inhibitors [28]. The TGF-*β* signaling pathway is controlled by many factors, including histone modification and epigenetic chromatin marks, such as histone H3 lysine methylation in TGF-*β*1-induced gene expression in rat mesangial cells under normal and high glucose conditions. TGF-*β*1 has been shown to increase the expression of ECM-associated genes, the connective tissue growth factor collagen-*α*1, and plasminogen activator inhibitor-1. Increased levels of histone H3 K4 methylation associated with active genes and decreased levels of histone H3 K9 methylation at these gene promoters accompany changes in expression [29]. TGF-*β*1 also increased the expression of H3 K4 methyltransferase SET7/9 and recruitment to these promoters. SET7/9 gene silencing with siRNAs significantly attenuated TGF-*β*1-induced ECM gene expression [9]. In this study, we did not examine changes in the mRNA levels of uH2A and uH2B as a consequence of uH2A and uH2B proteins stimulation by high glucose. This implies that there is no difference in the gene order of histones H2A and H2B, except for posttranslational modifications, including histone ubiquitination. We observed that the mRNA level of TGF-*β* dramatically increased followed by changes in uH2A and uH2B proteins. In summary, changes in uH2A and uH2B protein expression induced by high glucose in GMCs may enhance the activation of TGF-*β* and influence the pathogenesis of DN.

A recent study reported that the ubiquitin proteasome inhibitor MG132 has an antifibrotic function. MG132 exerts an antifibrotic effect by simultaneously downregulating type I collagen and a tissue inhibitor of metalloproteinase-1 and upregulating metalloproteinase-1 production in human dermal fibroblasts [30]. Tubular injury in a rat model of type 2 diabetes was shown to be prevented by MG132 by reducing renal tubule interstitial fibrosis [31]. Several studies have shown that MG132 has an effect on mitigating renal fibrosis by inhibiting the expression of kidney fibronectin mRNA in rats with early diabetic nephropathy and could improve proteinuria and other symptoms [32]. 

Research on histone ubiquitination is scarce, and inhibitors that can effectively and specifically block the ubiquitination of histones have not been described. The process of histone ubiquitination is similar to the ubiquitination of other proteins. MG132 is a specific ubiquitin proteasome inhibitor that can inhibit activation of the TGF-*β* signaling pathway, which is important in the development of fibrosis in DN [33]. However, there is not any evidence in the literature about whether MG132 can inhibit histone ubiquitination disorders or eliminate epigenetic metabolic memory to treat DN. Our experiments show that disorders involving histone H2A and H2B ubiquitination can exhibit an apparent reversal trend based on treating rat glomerular mesangial cells with MG132 and 30 mmol/L high glucose. After eliminating the dysfunction of histone ubiquitination, the expression of TGF-*β* mRNA was inhibited following MG132 intervention. This suggests that ubiquitin proteasome inhibitors may have a positive function in the treatment of diabetic nephropathy by inhibiting the disorders involving histone H2A and H2B ubiquitination that affects gene expression of TGF-*β*.

The proteasome inhibitor MG132 induces apoptosis. For example, MG132 inhibited the PI3K/Akt and NF*κ*B pathways, promoted mitochondrial depolarization, and decreased the concentration of mitochondrial antiapoptotic protein. MG132 also mediated activation of p38-JNK1/2 signaling and enhanced selective apoptosis in glioblastoma cells [34, 35]. MG132 is promising for cancer treatment because it markedly inhibited the growth of malignant tumor cells and arrested cells in the G2/M phase of the cell cycle, and the cells become apoptotic [36]. Strom and Panlsen reported that MG132 inhibited the ubiquitin proteasome, which degrades the apoptosis inducer NGFI-B and is a nuclear receptor [37]. Thakur suggested that MG132 inhibits histone deacetylation by increasing the degradation of histone deacetylase induced by green tea polyphenols in a prostate cancer cell line, followed by cell arrest and apoptosis [38]. Therefore, the ubiquitin proteasome inhibitor MG132 could inhibit the proteasome and induce apoptosis. Possible answers regarding the causes of histone ubiquitination were identified in this study, but the detailed mechanisms involved will require additional research. Determining the specific inhibitors of histone ubiquitination and discovering the role of histone ubiquitination in diabetic nephropathy renal fibrosis remain a challenge.

In conclusion, we demonstrated that the high glucose may induce the ubiquitination of histone H2A and reduce the ubiquitination of histone H2B in GMCs. The changes of histone ubiquitination in GMCs could activate TGF-*β* signaling pathway involved in the pathogenesis of diabetic nephropathy.
